# Supplementary material for: Automated identification of sedimentary structures in core images using object detection algorithms
Source: PLoS One. 2025 Jul 18;20(7):e0327738. doi: 10.1371/journal.pone.0327738 (PMC12273977; doi:10.1371/journal.pone.0327738)
Supplement: Attachment — Submitted filename: Rebuttal Letter.docx [file pone.0327738.s005.docx]

Rebuttal Letter

**Dr. Przemysław Mroczek**
Academic Editor
PLOS ONE

**Dear Dr. Mroczek,**

We appreciate the time and effort the editorial team has taken to review our manuscript **PONE-D-25-07311**, titled *"Automated Identification of Sedimentary Structures Using Object Detection."* We have carefully addressed the concerns raised regarding data availability and research transparency, and we outline our responses below.

**1. Data Availability Statement**

**Reviewer’s Comment:**
*"The manuscript does not fully comply with PLOS ONE’s policies on data availability. The current statement does not provide sufficient justification for access restrictions."*

**Response:**
To ensure compliance with **PLOS ONE’s policies on data availability**, we have made all relevant data publicly accessible through our **GitHub repository**. Due to the size of the dataset and limitations in the journal's submission system, the dataset is not included as a supporting file. Instead, the data required to reproduce our results is now openly available at the following link:
 <https://github.com/amarjuma2010/Object-detection-for-sedimentary-structures>

This repository includes data summaries and details on how to access the dataset.

**2. Code Availability & Research Reproducibility**

**Reviewer’s Comment:**
*"There is no reference to a publicly accessible repository for the code used in the analyses. Given that the study employs deep learning models (YOLOv4 and Faster R-CNN), it is essential that the scripts, configurations, and implementation details be made available for reproducibility."*

**Response:**
We have now publicly shared the inference code in a GitHub repository:
 <https://github.com/amarjuma2010/Object-detection-for-sedimentary-structures>

This repository includes:

- **Inference scripts** for running predictions on new images and videos.
- **Pre-trained model weights** for both YOLOv4 and Faster R-CNN.
- **Instructions for using the inference pipeline to replicate our results.**
- **Predicted videos demonstrating model performance** in the predicted_videos/ directory.

Due to GitHub's file size handling limitations, the predicted videos can be downloaded by cloning the repository using the following command:

git clone <https://github.com/amarjuma2010/Object-detection-for-sedimentary-structures.git>

or: !git clone https://github.com/amarjuma2010/Object-detection-for-sedimentary-structures.git

The repository's **README file provides detailed instructions** for accessing and utilizing the code and videos. This approach ensures full reproducibility and transparency while accommodating the journal’s file size constraints.

Additionally, we have updated the **Methods section** in the manuscript to explicitly cite this GitHub repository and provide detailed descriptions of how it can be used to reproduce the results. The following two paragraphs have been added in the tracked changes version (p11-12):

*"The inference codes for automated identification of sedimentary structures using YOLOv4 and Faster R-CNN models are available in this publicly accessible GitHub repository:* [*https://github.com/amarjuma2010/Object-detection-for-sedimentary-structures*](https://github.com/amarjuma2010/Object-detection-for-sedimentary-structures)*. The repository includes pre-trained model weights, inference scripts, and instructions for running predictions on new images and videos. This ensures full reproducibility of the object detection process applied in this study."*

*"The inference code is capable of generating predictions on both images and videos, producing annotated results with detected bounding boxes, class labels, and confidence scores. The predicted videos demonstrating the model's performance are included in the attached GitHub repository."*

We believe these revisions address all the concerns raised and ensure that our manuscript meets **PLOS ONE’s requirements for data availability and research transparency**. We appreciate the opportunity to improve our submission and look forward to your feedback.

**Sincerely,**
Ammar J. Abdlmutalib
Corresponding Author
**King Fahd University of Petroleum & Minerals**
Email: ammar.mohammed@kfupm.edu.sa
