# Supplementary material for: Automated identification of sedimentary structures in core images using object detection algorithms
Source: PLoS One. 2025 Jul 18;20(7):e0327738. doi: 10.1371/journal.pone.0327738 (PMC12273977; doi:10.1371/journal.pone.0327738)
Supplement: Attachment — Submitted filename: Responses to Reviewers.docx [file pone.0327738.s006.docx]

**Response to the Editor and Reviewers**

Dear Dr. Mroczek,

We sincerely thank you for your detailed editorial assessment and for overseeing the review of our manuscript, *“Automated identification of sedimentary structures using object detection.”* We would also like to express our gratitude to both reviewers for their thoughtful comments and constructive feedback, which have helped us improve the clarity and presentation of our work. We appreciate the positive reception and the recognition of the study’s scientific contribution and methodological robustness.

Below, we provide a detailed point-by-point response to the comments from both the reviewers and the editor. We have revised the manuscript accordingly and believe these changes have strengthened its content and clarity.

**Response to the Editor’s Comments**

**Title suggestion:** The editor suggests more specific titles for clarity and search optimization.

**Response:** We appreciate this suggestion. We have revised the title to:
**“Automated identification of sedimentary structures in core images using object detection algorithms”**

**Keyword revision:** Diversify keywords and avoid repeating terms from the title.

**Response:** Updated. The new list of keywords includes:
*Core image analysis, convolutional neural networks, facies recognition, supervised learning, sediment core interpretation, deep learning, lithofacies detection*

**Abstract revisions:** Streamline and provide broader geological context.

**Response:** Revised. We have rewritten the abstract to remove numerical training split details, provide a clearer geological scope (siliciclastic environments), and consolidate redundant phrases in the conclusion. These revisions were added to page **2** of the track-changes version of the manuscript (lines 20-35).

**Classification metrics repetition:** Remove redundancy in definitions.

**Response:** Revised. The metrics section has been rewritten for conciseness, with clear but non-repetitive definitions of accuracy, precision, recall, F1-score, IoU, and mAP. These revisions were added to pages **10-13** of the track-changes version of the manuscript (lines 199-239).

**Clarification of Split-I phrasing:** Indicate that training and validation were performed on distinct subsets.

**Response:** Corrected. We now clearly state that Dataset 1 was partitioned into separate, non-overlapping subsets for training and validation in Split-I. These revisions are found in page **6** of the track-changes version of the manuscript (lines 112-114).

**Repeated phrasing (“greater time efficiency”):** Harmonize language around YOLOv4’s speed advantage.

**Response:** Addressed. We revised repetitive phrases throughout the text to harmonize descriptions of YOLOv4’s computational efficiency, replacing redundant expressions with consistent terminology such as “faster processing” and “reduced inference time”. This issue was addressed in accordance to the editor’s suggestion in abstract, discussion, and conclusion. These revisions are found in page **33** of the track-changes version of the manuscript (lines 652-655).

**Citation formatting and “e.g.” usage:** Ensure consistency and appropriate placement of URLs.

**Response:** Revised throughout. We have standardized citation formatting, corrected the usage of “e.g.,” and moved all URLs from in-text citations to the Data Availability Statement or reference list. These revisions are found in page **38-39** of the track-changes version of the manuscript (lines 771-778).

**Detailed panel references in Figures 12 and 13:** Move detailed panel descriptions to figure captions.

**Response:** Completed. We revised the main text to provide general summary insights and relocated specific panel descriptions to the figure legends. These revisions are found in page **27-28** of the track-changes version of the manuscript (lines 528-536) and page 29 (lines 558-565).

**Response to Reviewer 1**

**Comment:** The reviewer appreciates the technical quality of the manuscript and notes that it provides a first-order approximation of facies analysis, recognizing that future work could explore more complex environmental reconstructions and textural classifications.

**Response:** We thank the reviewer for their supportive feedback and insightful observations. We have now addressed these suggestions in the *Discussion* section, explicitly acknowledging that the study represents a foundational step and outlining directions for future developments. These include potential extensions to analyze clast textures, bioturbation complexity, and depositional sequences, as well as integrating organic indicators and three-dimensional analysis methods. These revisions are detailed in the *Discussion* section, under the subsection titled **“Model implications and application potentiality”** (page 36 of the track-changes version).

**Response to Reviewer 2**

**Comment on full URLs (lines 104, 109, 125):** The use of full website addresses in the main text should be avoided.

**Response:** Addressed. All full URLs have been removed from the main text and relocated to the **Data Availability Statement** or cited properly in the **References** section, following journal guidelines. These revisions can be found on pages **38–39** of the track-changes version of the manuscript.

**Comment on line 123 – Figure 1c:** More emphasis should be placed on the data processing steps shown in the workflow diagram.

**Response:** Revised. We have expanded the accompanying description in the text to provide a clearer summary of the image processing workflow illustrated in Figure 1c. The revised passage now explains the sequence from data collection and annotation through augmentation, training splits, and model evaluation. These revisions can be found on pages **6–7 (lines 123-129)** of the track-changes version of the manuscript.

**Comment on line 127 – Imaging variability and 3D potential:** The authors should address the variability in lighting and the possibility of capturing 3D perspectives.

**Response:** Clarified. We have revised the text to mention the varying lighting conditions during image acquisition and acknowledge the potential for rotating cores to capture multi-angle or pseudo-3D images. We also note this as a possible avenue for future enhancement of automated analysis. These revisions were added to pages **36–37** of the track-changes version of the manuscript (lines 727-732).

**Comment on line 159 – Double citation:** The manuscript includes duplicate citations.

**Response:** Corrected. All instances of double citation have been reviewed, and formatting has been revised to ensure consistency and adherence to journal style.

**Comment on structural feature diversity and error rates:** The reviewer notes that the model performed best with morphologically distinct features, and error rates increased when structures were similar.

**Response:** We agree and have incorporated this observation into the *Discussion* section, specifically under the “Three Splits of YOLOv4” subsection. We highlight how structural similarity posed challenges for the model and suggest ways future work might improve discrimination in such cases. These revisions were added to page **32** of the track-changes version of the manuscript (lines 632-642).

We have uploaded a revised version of the manuscript reflecting all of the above changes, and we hope that it now meets the publication standards of *PLOS ONE*. We sincerely thank you again for the opportunity to revise and improve our work.

Kind regards,
**Ammar J. Abdlmutalib**
On behalf of all authors
College of Petroleum Engineering & Geosciences, King Fahd University of Petroleum & Minerals, Dhahran 31261, Saudi Arabia
[ammar.mohammed@kfupm.edu.sa](mailto:ammar.mohammed@kfupm.edu.sa)
