# Supplementary material for: Automated identification of sedimentary structures in core images using object detection algorithms
Source: PLoS One. 2025 Jul 18;20(7):e0327738. doi: 10.1371/journal.pone.0327738 (PMC12273977; doi:10.1371/journal.pone.0327738)
Supplement: Attachment — Submitted filename: Response to the additional editor comments.docx [file pone.0327738.s007.docx]

**Editor Comment 1: Expert Validation**

**“The study lacks any mention of expert validation of the image annotations or the predicted classifications…”**

**Response to Editor:**
We appreciate the editor's attention to this critical methodological aspect. We now clarify in the revised Dataset (page 6, lines 108-114) and Discussion (page 36-37, lines 710–716) that expert validation was an integral part of both the annotation process and the interpretation of model outputs. Domain experts, including sedimentologists with expertise in ichnology and clastic sedimentology, were directly involved in labeling the core images and in defining classification criteria. Their input ensured consistency, geological relevance, and accuracy across the labeled dataset. This validation process underpins the credibility of the model’s performance metrics and addresses the concern about label quality.

**Editor Comment 2: Class Imbalance**

**“The authors do not discuss how they addressed the clear class imbalance in their dataset…”**

**Response to Editor:**
Thank you for raising this point. We have elaborated in the Discussion (page 34-35, lines 659–679) on the natural origins of class imbalance in our dataset, which stems from the depositional environments represented—dominated by siliciclastic deep-marine and deltaic facies. Consequently, features like bioturbated media and mud drapes are overrepresented, while others like wavy bedding and soft-sediment deformation are rarer. Standard oversampling was avoided to prevent overfitting via synthetic redundancy. Instead, we employed augmentation techniques—specifically brightness, blur, cropping, and exposure adjustment—which boosted model performance by nearly 30%. Furthermore, we attempted reducing class counts from 19 to 13 to mitigate imbalance while preserving critical sedimentary variability. The latter strategy worsened the performance. In addition to dataset curation and targeted augmentation strategies, we implemented class weighting during the training of both YOLOv4 and Faster R-CNN models. This technique assigned higher weights to underrepresented classes to ensure balanced learning and minimize bias toward more abundant structures. The inclusion of class weighting contributed to improved detection consistency across rare and frequent sedimentary features, reinforcing the robustness of our classification framework.

**Editor Comment 3: Misclassification Errors**

**“The discussion of misclassification errors remains superficial… A focused paragraph discussing the likely causes of common errors… is necessary…”**

**Response to Editor:**
Thank you for highlighting the need for a more in-depth analysis of model misclassifications. In response, we have added a focused paragraph in the Discussion section (lines 529-539, pages 28) analyzing sources of confusion, particularly between morphologically overlapping or transitional structures. For example, mud drapes were frequently confused with bioturbated sandy/muddy media when appearing as disconnected features in flaser laminations or as connected mud layers that are selectively disrupted by bioturbation, or with thin massive mudstone beds when appearing as continuous layers within wavy bedding. Similarly, conglomerates were occasionally misclassified as massive sandstone under low-resolution conditions. These issues stem from intrinsic visual similarities and imaging limitations. The revised paragraph discusses how these ambiguities arise despite well-defined sedimentological criteria and outlines future solutions, including the incorporation of stratigraphic context and enhanced imaging or model architectures. This addresses the editorial concern by demonstrating a critical understanding of the model’s limitations and informing future development strategies.

**Editor Comment 4: Class Representation per Split
“It remains unclear whether all structure classes were present in the test sets of each data split...”**

**Response:**
We thank the editor for this critical observation. To ensure transparency and clarify class distribution, we have now included Supplementary Tables S1–S3, which provide a detailed breakdown of the number of bounding boxes for each structure class across the training and test sets for Splits I, II, and III. These tables confirm that all 15 classes were represented in each split, ruling out the absence of classes as a cause for performance decline in Split III. A reference to these tables has been added to the manuscript in Section 4 (Discussion), where we discuss split-specific performance variability. See (page 29, lines 555–558).

**Editor Comment 5: Inference Time**

**“It is essential that the authors report inference time for the Faster R‑CNN model…”**

**Response:**

**Revised Response to Editor (Inference Benchmarking)**

We thank the editor for this important observation. In the *Results* section (page 24, lines 458–464), we have now included inference benchmarking for both models under identical conditions (batch size = 32). Specifically, Faster R-CNN processed 10 test images in approximately 25.0 seconds (average ~2.5 s/image), whereas YOLOv4 required approximately 32.0 seconds (average ~3.2 s/image). This direct comparison now clearly demonstrates YOLOv4’s slower inference in our setup, which contrasts with common expectations for a one-stage detector and underscores the importance of framework-specific implementation details.

In the *Discussion* section (“One-stage vs two-stage detection”), we expanded the discussion to highlight that while YOLOv4 is theoretically expected to be faster due to its one-stage architecture and optimized Darknet backbone, real-world performance can diverge depending on the software framework, hardware utilization, and post-processing strategies. In contrast, Faster R-CNN’s more stable and mature implementation in the PyTorch ecosystem may explain its relatively faster inference in our tests. These findings emphasize the necessity of conducting benchmarking under consistent, real-world conditions, as actual runtime performance may differ significantly from theoretical assumptions.

We also now cite two relevant studies [48, 49] that have reported similar results, where YOLOv4 (or relevant Darknet-53 extractor) demonstrated slower inference performance than Faster R-CNN (or relevant ResNet-50 extractor) in certain contexts, further supporting the observed discrepancy.

**Editor Comment 6: Classification Metrics Section Too Lengthy:
“The section describing classification metrics is disproportionately long and remains overly didactic. While the inclusion of metric definitions is acceptable, the full mathematical formulas may be better suited to supplementary material, allowing the Methods section to remain focused and concise.”**

**Response:**
We appreciate the editor’s suggestion and have revised the Classification Metrics subsection in the Methods (page 10-11, lines 197-204) to improve clarity and conciseness. While retaining essential definitions for precision, recall, F1-score, Intersection over Union (IoU), and mean Average Precision (mAP), we have relocated the full mathematical formulas and extended explanations to the Supplementary Materials (Table S4). This adjustment ensures that the main text remains focused and accessible, in line with journal expectations for readability and methodological clarity.

**Editor Comment 7:
“Moreover, the process of image annotation and class distinction requires clarification. The ms must specify whether the distinction between visually similar classes (e.g. mud drapes vs bioturbated muddy media) was based on consistent quantitative thresholds, expert visual assessment, or a combination of both. The current description remains vague and limits reproducibility.”**

**Response to Editor:**
We appreciate the editor's request for clarification. The manuscript has been revised to explicitly describe the annotation protocol used for visually similar classes. In Discussion section (“Challenges Related to Labeling Inconsistency”) (page 33, lines 635-642), we now state that particular attention was given to abundant and frequently misclassified classes, such as mud drapes and bioturbated media. These were consistently annotated using sedimentological criteria informed by expert visual assessment. Specifically, mud drapes were identified as fine-grained, laterally continuous to semi-continuous layers recurring in heterolithic facies, while bioturbated media were characterized by irregular disruption and burrow textures in muddy or sandy matrices. These distinctions were guided by domain expertise to ensure reproducibility and minimize ambiguity. Additionally, we refer to this approach in the “Sedimentary Structures” subsection, reinforcing the methodological rigor of the labeling process.

**Editor Comment 8: Lack of formal comparison with prior work (e.g., Zhang 2021)
“Despite the claim that this is the first large-scale application of object detection to sedimentary structures, no formal comparison with previous studies is provided. The authors are required to include a summary table comparing their dataset, class range, and performance metrics with prior works, including Zhang 2021 and studies applying CNNs to lithofacies identification.”**

**Response to Editor:**
We appreciate this valuable comment. To contextualize the contribution of our study within the existing literature, we have now included a direct comparison with Zhang et al. (2021), one of the only prior studies applying CNNs to sedimentary structures. The comparative summary (now provided in Table 6) outlines differences in dataset scale, class diversity, validation strategy, model type, and output granularity. We also integrated a concise synthesis of this comparison in the opening paragraph of the Discussion section (page 26, lines 490-499), where we highlight how our work extends prior efforts through the use of object detection, multi-class bounding box annotations, and structured split validation. This addition helps underscore the novelty and scale of our approach relative to earlier classification-based studies.

**Top of Form**

**Editor Comment 9: Figure Captions:
“In addition, the figure captions should be carefully revised and substantially shortened. Several current captions (e.g. Figures 5 to 13) are excessively detailed and contain interpretative content or repetitions of information already presented in the main text. Figure legends should be limited to concise descriptions of what is shown...”**

**Response to Editor:**
We thank the editor for this constructive feedback. In accordance with the journal’s guidelines, we have carefully revised the captions for Figures 5 to 13 to ensure brevity and clarity. All interpretive content, extended model comparisons, and data analysis previously embedded in figure legends have been relocated to the Results or Discussion sections as appropriate. The revised captions now succinctly describe each figure and its panels, maintaining only essential explanatory information (e.g., class names or key identifiers) necessary for visual interpretation. This streamlining ensures consistency with journal standards and enhances the manuscript's readability.

**Bottom of Form**

**Editor Comment 10: Language Clarity and Redundancy:**
**“Finally, while the English language is generally clear, there are residual issues with fluency, repetition, and phrasing. Phrasal redundancy (e.g. ‘the model demonstrates ability to learn’ or ‘predictions show confusion between…’) appears frequently and should be eliminated. A thorough proofreading for academic tone and conciseness is required.”**

**Response to Editor:**
We thank the editor for this helpful comment. In response, we have thoroughly revised the manuscript to eliminate phrasal redundancies, improve fluency, and ensure a concise and academically appropriate tone throughout. *This has been applied in the clean copy not in the track-changes copy*. Particular care was taken to revise expressions such as “the model demonstrates ability to learn” and “predictions show confusion between…” along with similar instances, aligning the text with the journal’s standards for clarity and style.
